# Acetabular revision using trabecular titanium (Delta TT) revision cups: A retrospective case series

**DOI:** 10.1051/sicotj/2022049

**Published:** 2022-12-21

**Authors:** Ahmed K. El Ghazawy, Ayman Abdelaziz Bassiony, Haytham Abdelazim, Saleh Gameel

**Affiliations:** Department of Orthopedics surgery, Faculty of Medicine Ain Shams University Cairo 11566 Egypt

**Keywords:** Acetabular revision, Acetabular defects, Trabecular titanium, Revision cups, Hip arthroplasty

## Abstract

*Background*: The annual rate of primary THA has been increasing with new designs promoting THA in the younger population, therefore increasing rates and complexity of hip revision surgeries. Different types of acetabular defects in hip revisions, usually make the use of primary cementless cups quite difficult. In complex defects, using cages with cemented cups or combining cementless cups with metal augments, are possible reconstruction solutions. The Delta TT acetabular revision system provides a solution to complex defects combining the advantages of both cage construct and primary implants, with modularity that helps restore anatomical hip centre and biomechanics. The aim of this study is to evaluate the short-term results of the use of the Delta TT revision system in acetabular revision surgeries. *Type of the study*: A retrospective case series. *Methods*: 24 patients underwent acetabular revision using (Delta TT) revision system, from 2018 to 2021. The mean follow-up was 20.75 months. Clinical and functional outcomes were assessed using Harris Hip Score. *Results*: The use of the Delta TT revision system in acetabular revision surgery provided adequate pain relief, and early patient mobilization. The preoperative HHS mean of 29.88 improved to a mean of 85.21, at the last, follow-up. None of the patients developed periprosthetic infection or loosening or nerve palsy during the follow-up period. *Conclusion*: Short-term clinical outcomes for the use of the Delta TT revision cup system in acetabular revision are encouraging with good functional outcomes and patient satisfaction.

## Introduction

For several years, total hip arthroplasty has been considered the gold standard solution for the management of many end-stage hip joint pathologies providing satisfactory clinical outcomes and long-term survival [1, 2]. It is considered a safe and cost-effective intervention [3]. The continuous increase in the number of THA surgeries, leads to an increase in the number and complexity of revision surgeries [4].

Management of severe acetabular bone loss in revision surgeries is considered a great challenge. The most important causes of large acetabular defects and severe bone loss include, periprosthetic infection, aseptic loosening, and wear osteolysis, or periprosthetic fractures [5]. Several classification systems have been used for grading acetabular defects as proposed by D’Antonio et al. [6] or Paprosky et al. [7].

The aim of actebular reconstruction is to fill the present defect with the restoration of the anatomical cup position [8]. The choice of the optimum acetabular reconstruction technique is influenced by many factors, the most important is the severity of bone stock loss together with the remaining host bone quality. Management of small contained cavitary defects can be done using impaction grafting with morselized bone or small strut graft with the use of a cementless primary hemispherical cup [9].

However, management of major defects requires the use of large structural grafts [9] in combination with large jumbo cementless cups [10] which usually provides excellent clinical outcomes. Other reconstruction methods include the combination of bone grafting together with antiprotrusio cages as the Burch-Schneider ring or Kerboull reinforcement plate and a cemented cup, usually providing an adequate clinical outcome [11, 12].

In the last 2 decades, the introduction of new acetabular components designed from highly porous materials greatly improved the outcomes of hip revision surgeries and is considered an important advance in hip surgery [9].

Titanium and its alloys were recently introduced for use in surgical purposes worldwide; titanium-based metals are used in the manufacture of dental implants, as well as plates and screws. These materials are also recently used in different orthopaedic surgeries for their favourable biomechanical properties, chemical stability, and biocompatibility [10].

Recently, trabecular titanium (TT) was introduced, which is a highly porous material with a structure that closely simulates the morphology of trabecular bone, providing desirable mechanical and osteoconductive properties [11, 13].

The aim of implant selection in acetabular revision is to achieve primary stability with long-term survival. As a result of the increased failure rates of reconstruction rings and cemented cup construct, a new system for acetabular revision was recently introduced for the management of acetabular bone loss in revision cases. The Delta Revision TT cup (LimaCorporate, Villanova di San Daniele del Friuli, Italy) is an acetabular component for revision cases, manufactured from trabecular titanium (TT), combining the advantages of a hemispherical primary cementless acetabular cup and those of a reconstruction cage [14].

To our knowledge, there is limited information in the current literature regarding the outcome of the Delta TT revision system in acetabular revision surgeries in comparison with other older reconstruction methods. This study aims to evaluate in our group of patients, the short-term results of the use of the Delta TT revision cup system in the management of acetabular defects in revision total hip arthroplasties.

## Materials and methods

*Study population*: The study included 24 hips in 24 patients with a failed acetabular component in previous THA with or without stem failure. Reconstruction was done using Delta TT acetabular revision system, over 4 years from 2018 to 2022. The mean follow-up was 20.75 months (range from 14 months to 30 months).

*Age gender and diagnosis*: The study group consisted of 6 females and 18 males, the mean age was 56 years (range 30–67). The indications for revision were: aseptic loosening (19 patients, 79.2%), septic loosening (re-implantation after two-stage exchange) (3 patients, 12.5%), and acetabular erosion following hemiarthroplasty (2 patients, 8.3%).

*Preoperative evaluation*: Full history and local clinical examination were taken, plain X-rays of the involved hip and pelvis and Ct scan for evaluation of acetabular bone defects. Preoperative laboratory investigations were done for assessment of the patient’s general condition and exclusion of periprosthetic infection (CBC, ESR and CRP quantitative titre). If both the CRP and the ESR are elevated, the hip should be aspirated preoperatively.

*Surgical technique*: A modified lateral approach was used for appropriate exposure, followed by extraction of the existing implant with preservation of acetabular bone stock as much as possible. After that bone defects were evaluated intraoperatively.

Bone defects were classified according to Paprosky classification and cases were graded as Paprosky type III defect (7 with III A and 15 with III B) and 2 cases were graded as type II B (Table 1).

**Table 1:** Distribution of the studied cases according to acetabular defects.

		No. = 24
Defect	2B	2 (8.3%)
	3A	7 (29.2%)
	3B	15 (62.5%)

After appropriate soft tissue debridement and removal of granulomatous tissue acetabular preparation was done with the use of reamers of different sizes. Morsellized cancellous bone graft was used to fill small cavitary defects, the graft was densely packed in the defects and impacted by reverse reaming.

Morcellised bone graft was used in 20 cases (83%). Metal augment was used in 19 cases (80%). For adjustment of orientation and to improve the stability of the implant a spacer was used in 10 cases (41%). Details are given in Table 2.

**Table 2:** Type of implant, spacers, bone graft and cranial augmentation, and liner surface data.

Variable	*n* (%)
Acetabular component	
TT revision	24 (100%)
Spacers	
Neutral	4 (17%)
+5 mm	2 (8%)
10°	0 (0%)
5 mm + 10°	1 (4%)
20°	1 (4%)
5 mm + 20°	2 (8%)
Bone graft	
Morcellised bone graft	20 (83%)
Augments	
12 mm	9 (37.5%)
18 mm	10 (41%)
Liner	
Polyethylene	19 (79%)
Ceramic	2 (8.3%)
Dual mobility	3 (12.5%)

Polyethylene liners were used in all cases, except for 2 cases in which ceramic liners were used and 3 cases in which a dual mobility articulation was used. The stem was replaced if loosening was detected radiological and confirmed intraoperatively which was needed in 19 cases, 80%. Extended trochanteric osteotomy (ETO) was performed in 10 cases (41%) which was reattached with metal cerclages.

**Figure 1:**
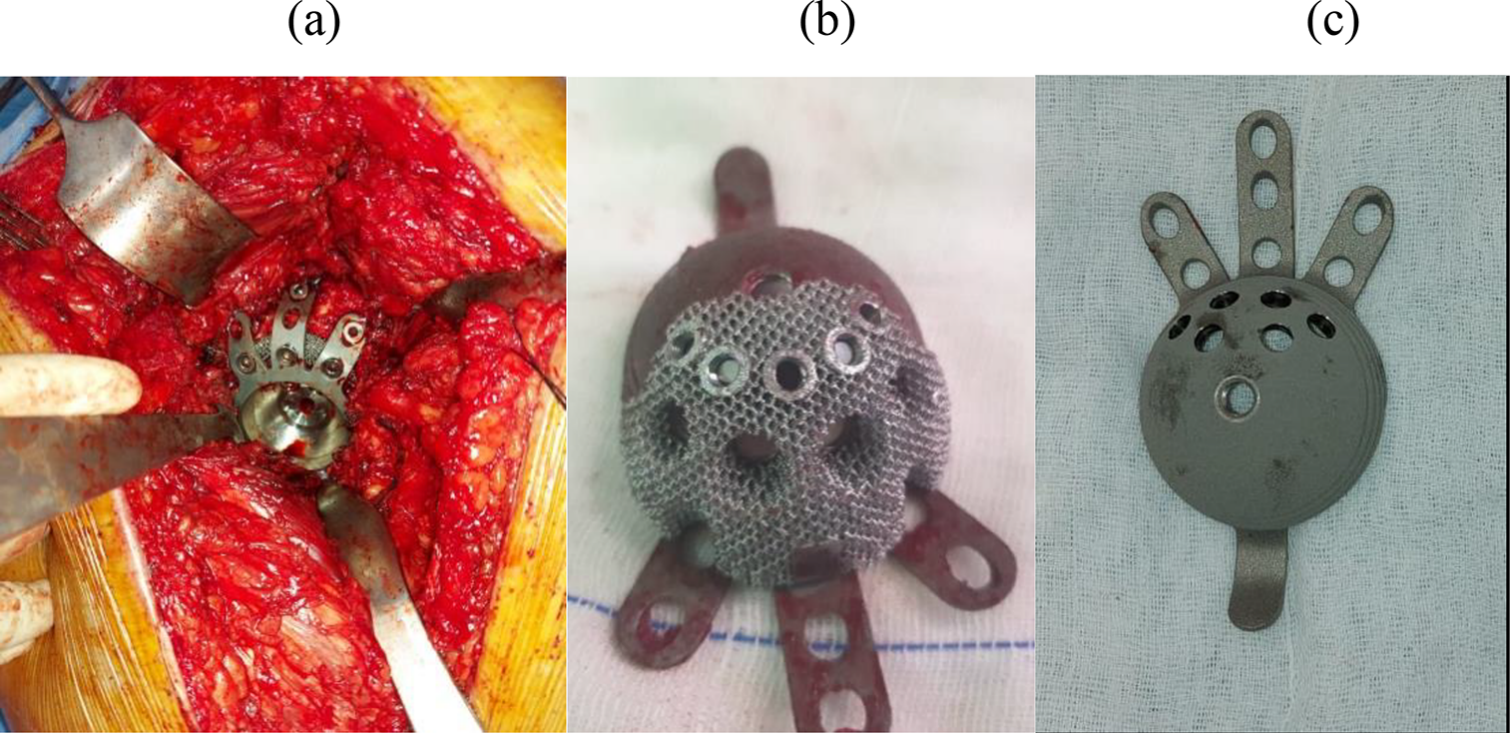
(a) Intraoperative image of the application of a TT revision cup with the addition of a hemispherical module. (b) Delta TT cup with augmenting fixed with screws (c) cup before augmenting application.

The Delta TT revision system (Lima Corporate, Udine, Italy) is a cage construct with 3 proximal side plates and a distal hook to provide extra construct stability and fixation, multiple screws can be applied through the holes of the side plates into the iliac bone. In our cases, the number of screws used in fixation was an average of 3 screws (range 2–4). A hemispherical pure TT augment can be applied if needed to the cup’s outer surface in presence of major defects (Figure 1).

To accommodate for the site and size of the present acetabular defect, the augment can be applied to the cup’s superior aspect in three different positions. Augment is secured to the cup using screws, without the need for cementation. This hemispherical augment is available in two different sizes, (12 mm and 18 mm).

**Figure 2:**
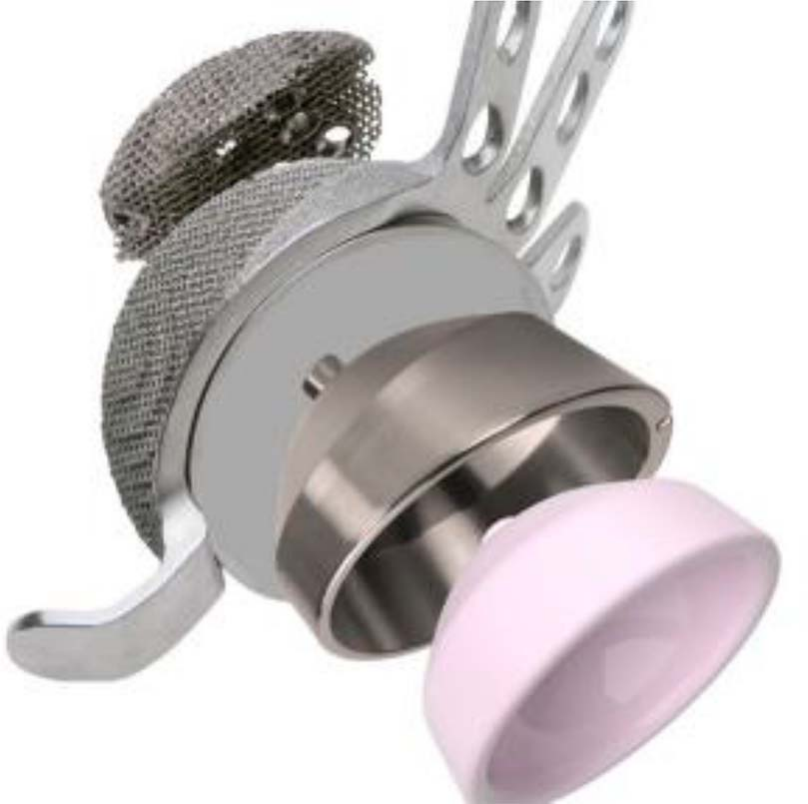
A figure showing the modularity of the cup and how to assemble the different components including the augment spacer and liner.

After augmenting fixation to the cup, the whole construct is applied in the position providing maximal host bone contact and coverage. A titanium spacer can be applied inside the cup for better adjustment of orientation and version if needed, (10° and 20° are available) and also may provide lateralization for the hip centre (Figure 2).

The femoral component revision was needed in 19 cases, indications included periprosthetic infection (*n* = 3), stem malposition in (*n* = 3), and aseptic loosening in (*n* = 13). Cementless long stems where used in all cases except one where the cemented stem was used.

*Postoperative protocol*: Patients were instructed to remain partially weight-bearing for 6–12 weeks depending on the clinical and radiographic assessment.

**Figure 3:**
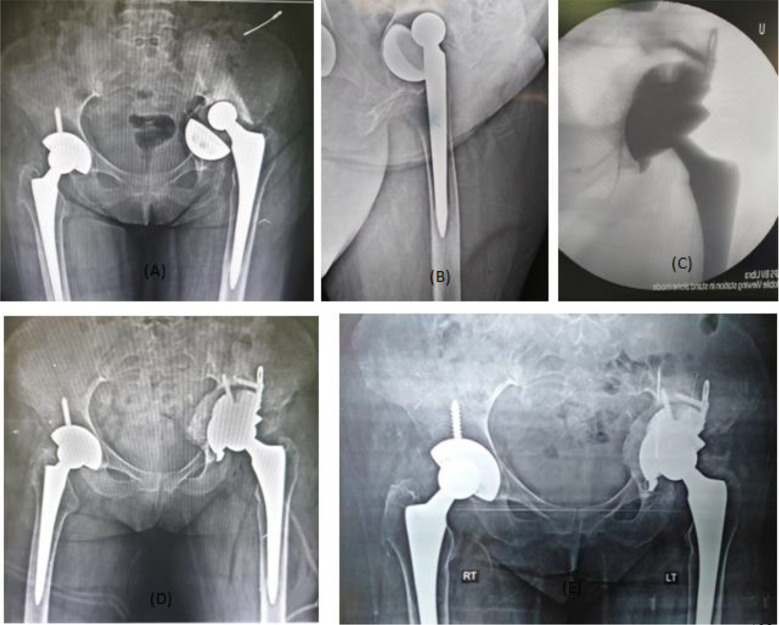
Male 50 years old with type IIIB defect treated with Lima TT revision shell and allograft. (A) Showing the preoperative X-ray AP view, (B) preoperative X-ray lateral view, (C) intraoperative image intensifier radiographs after cup application, (D) postoperative X-ray and (E) X-ray after 2 years shows allograft was completely incorporated with the host bone with stable reconstruction.

*Radiological evaluation*: Components position, radiolucency in DeLee and Charnley zones [15], graft-implant interface, and bone-bone graft interface were evaluated. Migration of implants was evaluated according to Hendricks and Harris criteria [16] and Onsten et al. [17], a change of implant position by more than 2 mm horizontally or vertically or a change of implant angle by more than 5° is considered radiographically significant. The radiolucencies at the bone-implant interface were evaluated according to DeLee and Charnley’s description [15], Radiolucent lines more than 2 mm were considered significant. Graft union, host bone-graft radiolucencies and signs of graft resorption were noted (Figure 3).

Bone graft incorporation was evaluated radiologically according to Gross [18] by bone trabeculae formation crossing the graft–host interface. Resorption was graded as minor (<1/3 of graft resorbed), moderate (1/3 to 1/2 of graft resorbed), and severe (>1/2 of graft resorbed).

*Clinical evaluation*: Harris Hip Score was used to evaluate the functional outcome. Postoperative complications including periprosthetic joint infection, aseptic implant loosening, or instability all were reported.

*Antibiotics prophylaxis*: A loading dose of 1st generation cephalosporin was given to all patients for antibiotic prophylaxis, and it was continued for 2 days postoperatively and then stopped if there was no fever or wound discharge.

*DVT prophylaxis*: Subcutaneous injection of a prophylactic dose of low molecular weight heparin (clexane) was used with all cases starting 12 h postoperatively and till discharge, then changed to oral anticoagulant as apixaban (eliquis 2.5 mg every 12 h) for 6 weeks.

**Figure 4:**
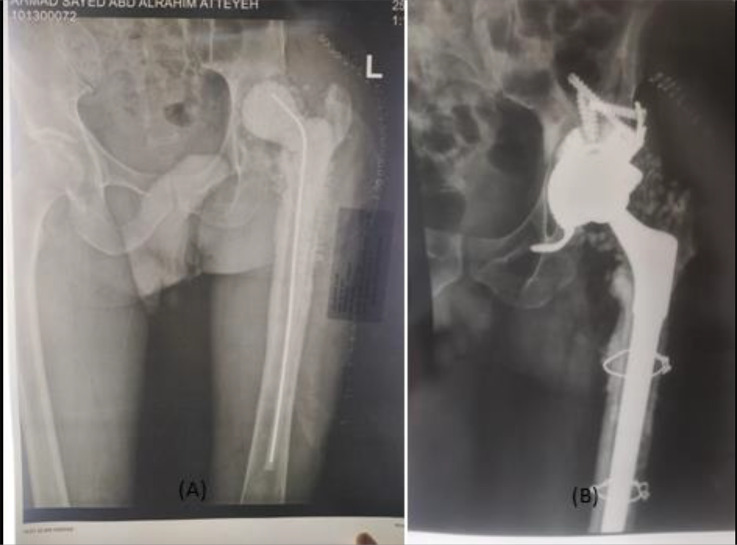
(A) preoperative X-ray for a case with hip spacer and acetabular defect, (B) postoperative X-ray after reconstruction using Delta TT revision system.

*Follow-up protocol*: Follow-up data were available for all cases, mean follow-up was 20.75 months (range from 14 months to 30 months). All patients were allowed partial weight bearing during the first 48 h postoperatively. Follow-up of wound dressing was done till stitches removal with no discharge or signs of infection except for mild serous discharge in one case, however frequent dressing and antibiotics were sufficient and did not require further intervention (Figure 4).

*Statistical analysis*: IBM SPSS statistics software, version 21 (IBM Corp., Armonk NY) was used for the analysis of the data. A paired sample *t*-test of significance was used when comparing related samples. A *p*-value was considered significant when <0.05 and highly significant when <0.001.

## Results

Till the last follow-up, the 24 hips showed good clinical and radiological outcomes, X-rays did not show any progressive radiolucency whether acetabular or femoral or change of implant position.

*Radiological results*: the hip centre was restored in 20 cases (83%) in the remaining 4 cases proximal migration of the hip centre of less than 6 mm was achieved. The cup inclination angle was an average of 40° (range 30–50). No change in cup position was found in any of the patients till the last follow-up.

**Figure 5:**
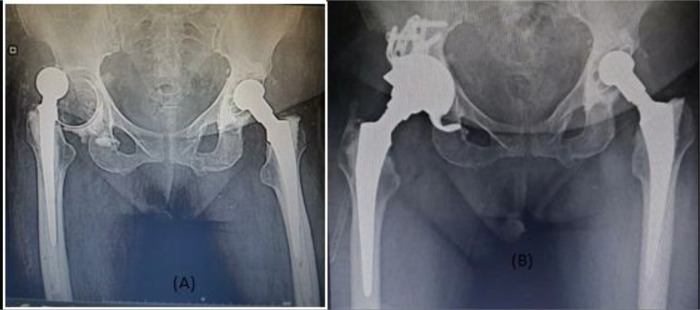
(A) Preoperative X-ray for a case with acetabular aseptic loosening, (B) postoperative X-ray after revision using Delta TT revision system with stem retention.

Bone graft showed full osteointegration in 18 of 20 cases where the graft was used in the reconstruction. Non-progressive radiolucency less than 2 mm in width was detected in one patient, this was in the junction of zone II and III and was graded as minor radiolucency according to Gross [18] Graft resorption was detected in another case however patient was satisfied clinically with no complaint (Figure 5).

*Clinically and functional results*: HHS improved from a mean of 30 (range 15–47) preoperatively to a mean postoperative score of 85 (70–98). With significant improvement in pain, function and mobility. According to the calculated *p*-value, there was a highly statistically significant difference between preoperative and postoperative HHS (Table 3). An LLD <1 cm was achieved in 20 hips (83%).

**Table 3:** Comparison between pre and post regarding HHS.

		Pre	Post	Test value	*P*-value	Sig
HHS	Mean+SD	29.88 ± 11.52	85.21 ± 8.70	−19.667*	0.000	HS
	Range	15 − 47	70 − 98			

* Refers to Paired *T* test.

*Complications*: None of our patients suffered from dislocation or nerve palsy, or periprosthetic fractures during the postoperative period. Also, there were no cases of DVT or pulmonary embolism or periprosthetic infection.

## Discussion

Management of acetabular bone loss in revision THA is challenging. There are several reconstruction techniques, however, the results are not always reliable [19]. This study reported our experience of an original reconstruction technique in acetabular reconstruction using TT revision cups in severe acetabular deficiency. In our study group, the results of this reconstruction technique were promising with good outcomes and functional improvement.

In our series, the TT revision cup demonstrated excellent fixation on the radiological evaluation at the most recent follow-up. All cups at the follow-up were well fixed without evidence of loosening or migration. We found a significant increase in the postoperative HHS functional score (from 30 to 85).

The limitations of our study were a limited number of patients, a short follow-up period, lack of comparison between the results of the use of the Delta TT system and other acetabular reconstruction options. These limitations should be addressed in future studies.

Reconstruction of acetabular defects should provide adequate primary fixation with durable construct. Options of reconstruction are numerous, the most frequently used in practice are; cemented cup with impaction grafting [20], hemispherical acetabular component [21], metal augments [22], ring and reconstruction cage [23], oblong components [24], cup-cage reconstruction [25], and custom triflange implants [26]. These options were used previously in our practice in the management of such complex cases, however, such defects are not frequently encountered.

The use of oblong revision cups limits the use of large structural allografts to deal with acetabular defects. Such reconstruction method showed good midterm clinical outcomes with good osteointegration and adequate primary fixation [27].

The introduction of the custom triflange acetabular components (CTAC) helped greatly in the reconstruction of large acetabular defects in revision cases with limited use of bulky structural grafting. However, limitations included time delay for customized manufacturing, high implant price and absence of modularity and limited possible bearing surfaces [28].

Acetabular components manufactured from TM have recently been a good option in the management of large acetabular defects with good survival rates in revision cases, as the idea of a cancellous porous metal surface promotes bone ingrowth and incorporation, also such material has preferable biomechanical properties [29].

In revision cases, reconstruction aims to achieve primary stability and long-term survival after adequate bone incorporation with cementless components, which usually provide better outcomes compared to cemented implants in such cases [30].

Recent studies showed that TM cups with or without the use of metal augments provide good survival rates (87–99.2%) the mean follow-up in these studies was 36–74 months [29]. Also systematic reviews showed lower rates of revision with TM implants in comparison with revision rings [18].

The bone graft should be partially unloaded and protected from high mechanical force during the process of remodelling. Recently, Makita described that bone graft osteointegration was completed in around 12 months in Paprosky type 3A and 3B bone defect [31]. The use of the Delta TT revision system protects the structural bone graft through the application of a screw to the pelvis, therefore partially unloading the grafted bone from excessive forces.

TT revision system when compared with custom-made cups has the advantage of being ready for application and its intra-operatively modularity. Revision TT shell is a good alternative to reconstruction cages in the management of major acetabular bone loss where the rim integrity is not enough to support the proper fitting of a hemispherical cup. This system has the advantage to provide adequate stability using the plates integrated into the cup. Besides the possibility of joint centre lateralization by using spacers giving this system a remarkable versatility helping to restore the anatomical hip centre of rotation, the lateral and longitudinal offset with less difficulty [32].

Trabecular titanium provided good results in complex primary arthroplasty cases. De Meo et al. [33] in a recent study using trabecular titanium in acetabular revision reported overall survival of the cup of 94.8% at a mean follow-up of (48.3 months). The authors reported a rate of aseptic loosening of 1.5% at 48.3 months.

Our results were comparable to Daniele Munegato et al., who performed 36 acetabular revisions in 34 patients using the Delta TT revision system, HHS improved from 40.5 preoperatively to 87, with no signs of implant loosening or mobilization during follow-up. The survival rate was 100% for aseptic loosening and 91.7% for any cause of revision [14] (Table 4).

**Table 4:** Results of recent studies.

Study	No. of patients	Mean follow up	Pre HHS	Post HHS	Graft incorporation	Loosening	Complications
Daniele et al.	36	39.8	40	87	60%	0	3
Perticarini et al	31	32		86.5		0	
Steno et al.	81	38.14			100%	0	3
Gallart et al.	67	30.5				2	6
Ceretti et al.	34	60	21	78.8	94%	1	2
Loris et al.	104	91	43.7	84	98%	1	16
Our study	24	20.75	30	85	96%	0	0

Also, Gallart et al. [34] in another study reported 67 revisions using Delta TT cups, the mean follow-up was 30.5 months, the rate of aseptic loosening was 2.9%, the infection rate was 4.5%, and the dislocation rate was 4.5%, these results are comparable however inferior to those of our study.

Another study by Steno et al. [28] performed 81 revision cases using the Delta TT system with a mean follow-up period of 38.14 months very good functional outcome was achieved in 53.8% of the cases and fair results in 12.5%. 3 cases with Type 3B defects showed cup cranial migration about 6 mm stabilized after 6 months. No signs of loosening or radiolucency were found till the last follow-up with good bone integration with the TT augment [28].

Also, Perticarini et al. [27] studied 31 revisions using the Delta TT system with a mean follow-up of 32 months, the postoperative mean HHS was 86.5. Implants were well fixed with no loosening or radiolucency during follow-up, and bone grafts were used in 24 cases with no radiological resorption.

Other reports have shown similar clinical outcomes and improvement in the functional scores after cup-cage reconstruction [35].

The clinical and radiological results of this study are comparable to other studies discussing this acetabular reconstruction technique. These results indicate that TT Revision System may be a very useful aid for surgeons facing challenging large acetabular defects. Furthermore, long-term studies are required to confirm these encouraging results.

## Conclusion

The use of Delta TT acetabular revision system may be considered as a good management option for major acetabular defects in difficult hip revision cases, providing adequate stability, promoting bone integration and its modularity helps restoration of anatomical hip biomechanics as much as possible with less difficulty.
